# Skin proteomic screening and functional analysis of differential proteins associated with coat color in sheep (*Ovis aries*)

**DOI:** 10.5713/ab.24.0014

**Published:** 2024-05-07

**Authors:** Zhihong Yin, Shitong Hao, Yuanyuan Zhao, Jinglong Li, Yunli Cui, Yaming Ge, Quanhai Pang

**Affiliations:** 1 College of Animal Science and Veterinary Medicine, Henan Institute of Science and Technology, Xinxiang, 453003, China; 2 Guizhou Provincial Key Laboratory for Biodiversity Conservation and Utilization in the Fanjing Mountain Region, Tongren University, Guizhou 554300, China; 3 College of Animal Science and Veterinary Medicine, Shanxi Agricultural University, Taigu, Shanxi 030801, China

**Keywords:** Coat Color, Pigmentation, Proteome, Sheep Skin

## Abstract

**Objective:**

Coat color is an important characteristic and economic trait in domestic sheep. In this study, we explored the potential mechanisms and the signaling pathways involved in coat color regulation for sheep.

**Methods:**

Isobaric tags for relative and absolute quantification (iTRAQ) technology was used to catalog global protein expression profiles in skin of sheep with black versus white coat color. Immunofluorescence was used to observe the expression localization of differential protein. Western blot and quantitative real time polymerase chain reaction (qRT-PCR) were used to evaluate their role in the coat color formation of sheep.

**Results:**

A total of 136 differential proteins were obtained in different coat colors, including 101 up-regulated and 35 down-regulated. Pigmentation function entries were enriched through gene ontology annotation. Tyrosine metabolism and platelet activation signaling pathway were extracted by Kyoto encyclopedia of genes and genomes analysis. Apolipoprotein A-1 (APOA1) and fibrinogen alpha chain (FGA) were found to be critical differential proteins by the interaction of differential proteins in the direct-interaction network diagram. Strikingly, twenty candidate differential proteins were screened, from which beta-actin (ACTB) protein showed higher expression in white sheep skin, while albumin (ALB), APOA1, MAOA (amine oxidase) and FGA proteins showed higher expression in black sheep skin, which was validated by immunofluorescence, western blot, and qRT-PCR.

**Conclusion:**

This study identified several novel proteins that may be involved in the coat color formation of sheep. The white and black sheep skin proteome profiles obtained provide a valuable resource for future research to understand the network of protein expression controlling skin physiology and melanogenesis in sheep.

## INTRODUCTION

Sheep are paramount wool-producing animals worldwide, and fiber color is a key trait contributing to the economic value of sheep. These traits were determined primarily by genetics [1] and the environment [2]. Sheep coat color is limited, and the most common types are white and black. However, compared with other colors, white fleece holds the greatest economic value because of its ability to be dyed any color. The natural colors of animal are increasingly gaining research interest due to the green revolution and consumer preference for natural products. Hence, factors that determine coat color in sheep are increasing a research hotspot.

In adult animals, coat color depends on the melanin produced and released by melanocytes present in the skin [3]. Melanocytes produce two types of melanin, namely, eumelanin (black to brown) and pheomelanin (yellow to reddish) [4,5], whose quality and ratio influence the coat color in hair follicle. Pigmentation was determined by gene action and gene interaction. Numerous genes have been found to affect coat color in some species, including sheep. For instance, melanocortin -1 receptor (*MC1R*) and c-Kit (c-Kit gene) are known to be major up-regulate and down-regulate genes of coat color in animals [6,7]; tyrosinase-related protein 1 (TYRP) and microphthalmia-associated transcription factor (MITF) are strong positional candidate gene in the melanogenesis pathway and contribute to color variation in mammals [8–10]. Research has been extended to microRNA that regulated coat color by deep sequence in the sheep [11] and goat [12]. Interestingly, skin transcriptome profiles are associated with coat color in sheep, revealing a host of genes including those known to exist in other animals, such as tyrosinase (TYR), MITF, and endothelin 3 (END3). Therefore, considerable knowledge on the molecular mechanisms and genetic regulation of coat color had been gained at the gene expression level (Fan et al [11]). However, the potential contribution of protein to differential expression associated with coat color has not been fully elucidated in sheep.

Proteomices has become an indispensable complement of transcriptome in life science, and as a powerfu Bicinchoninic acid l tool to investigate protein function and changes. Isobaric tags for relative and absolute quantitation (iTRAQ), which is based on the enzymatic digestion of proteins prior to isobaric labeling. iTRAQ is a superior choice in quantitative proteomics due to their high proteome coverage and labeling efficiency, and has been applied to various academic fields in the latest years [13–15]. In the present study, we investigated the biological functions of the differentially abundant proteins between white skin (WS) and black skin (BS) of sheep by using iTRAQ to unravel the mechanism of coat color. We provided new insights into the formation of coat color and valuable evidence to better understand the potential role of proteins regulating coat color at the proteomic level.

## MATERIALS AND METHODS

### Sheep skin sample collection

Skin samples were collected from sheep (Ovis aries) housed and maintained in accordance with the International Guiding Principles for Biomedical Research Involving Animals (https://www.cioms.ch/frame1985textsofguidelines.html) approval number of IACUC: LLSC2024038. Every effort was made to minimize the suffering and number of animals used in the study. The animals were locally anaesthetized with hydrochloridum (1.5 mL of 3%, i.h.), following the approval (reference number 2010 [088]) of the Animal Hospital of Shanxi Agricultural University to decrease animal suffering. Six healthy 1-year-old (weight: 50±5 kg) all-white and all-black females sheep (Dorper sheep♂×Mongolia sheep♀, three sheep per color) were selected from a sheep farm in Shanxi Agriculture University (Shanxi Province, China) for sample collection. Sheep are fed freely on the farm. The wool at the hindquarter of the sheep was carefully trimmed using fine dissecting scissors to avoid bleeding. Each piece of skin (8 mm in diameter) from the hindquarter was collected through punch skin biopsy under local anesthesia and immediately placed in liquid nitrogen. Six additional WS and BS tissues (three sheep per color) were fixed in Bouin's solution for 24 h at 4°C and then extensively washed in 70% ethanol.

### Protein extraction and digestion

Frozen skin tissues were ground into fine powder in liquid nitrogen and dissolved in 300 μL of SDT buffer (4% sodium dodecyl sulfate [SDS], 100 mM Tris-HCl, 1 mM dithiothreitol [DTT], pH 7.6). After incubating with boiling water for 15 min, ultrasonication (100 W, 10 times; 10 s per time with 15 s intervals), and incubation with boiling water for 15 min, the homogenate was centrifuged 14,000 g for 30 min. The supernatants were collected, and protein content was determined with bicinchoninic acid protein assay reagent.

Each group of samples (200 μg of protein) were placed in DTT (161-0404; Bio-rad, Hercules, CA, USA) such that the final concentration reached 100 mM. The samples were incubated with boiling water for 5 min, cooled to room temperature, added with 200 μL of UA buffer (8 M urea, 150 mM Tris-HCl, pH 8.0), transferred to 10 Kd ultrafiltration centrifuge tubes (Sartorius), and centrifuged for 15 min. The retained protein was washed with 200 μL of UA buffer, centrifuged and added with 100 μL of iodoacetamide (IAA) (50 mM IAA in UA) buffer to block reduced cysteine residues. The samples were incubated for 30 min in darkness and centrifuged under the above conditions. The filtrates were washed three times in 100 μL of UA buffer and added with 100 μL of dissolution (DS) buffer (AB SCIEX, Boston, MA, USA; 50 mM triethy lammoniumbicarbonate at pH 8.5) twice, followed by centrifugation for an additional 10 min under the same condition. The protein suspensions were digested with 7 μg of trypsin (Promega) in 40 μL of DS buffer and incubated at 37°C for 16 to 18 h. Finally, the peptides were collected by centrifugation, and peptide content was estimated using UV light at 280 nm.

### iTRAQ reagent labeling and peptide fractionation with strong cation exchange chromatography

Peptide labeling was performed according to the manufacturer's instructions of an iTRAQ Reagent-8plex Multiplex Kit (AB SCIEX, USA). For iTRAQ labeling, each iTRAQ reagent was dissolved in 70 μL of ethanol and added to the respective peptide mixture (40 μg), followed by multiplexing and vacuum driying. The iTRAQ aliquots were combined with peptide mixtures from six different samples, respectively, and incubated at room temperature for 1 h. The iTRAQ-labeled peptides were fractionated by strong cation exchange (SCX) chromatography by using the AKTA Purifier system (GE Healthcare, Boston, MA, USA). The peptide mixture was loaded onto a Poly SULFOETHYL 4.6×100 mm column (5 μm, 200 A; PolyLC Inc, Columbia, MD, USA) in buffer A (10 mM KH_2_PO_4_ in 25% of ACN, pH 3.0) and separated with a linear gradient of buffer B (10 mM KH_2_PO_4_ in 25% of CAN, 500 mM KCl, 500 mM KCl) for 2 min. With buffer A as equilibrium, buffer B was added in gradient for separation. The elution was monitored by absorbance at 214 nm, and fractions were collected every 1 min. The collected fractions (about 30 fractions) were finally combined into six pools and desalted on C18 Cartridges (Empore SPE Cartridges C18 (standard density), 7 mm bed I.D., volume = 3 mL; Sigma, St Louis, MO, USA). Each fraction was concentrated by vacuum centrifugation. All samples were stored at −80°C until liquid chromatograph mass spectrometer (LC-MS/MS) analysis.

### Liquid chromatography (LC) - electrospray ionization (ESI) Tandem mass spectrometer (MS/MS) analysis by Q Exactive

Each fraction sample was analyzed on a Q Exactive mass spectrometer (MS) system coupled to Easy nLC (Proxeon Biosystems, now Thermo Fisher Scientific, Waltham, MA, USA). About 10 μL of each fraction was injected for nano-LC-MS/MS analysis. The peptide mixture (5 μg) was loaded onto a C18-reversed phase column (Thermo Scientific Easy Column, 10 cm long, 75 μm inner diameter, 3 μm resin) in buffer A (0.1% formic acid) and separated with a linear gradient of buffer B (80% acetonitrile and 0.1% formic acid) at a flow rate of 250 nL/min controlled by IntelliFlow technology over 140 min. MS data were acquired using a data-dependent top 10 method by dynamically choosing the most abundant precursor ions from the survey scan (300 to 1,800 m/z) for higher-energy collision dissociation (HCD) fragmentation. The target value was determination based on predictive automatic gain control (pAGC). The dynamic exclusion duration was 60 s. Survey scans were acquired at a resolution of 70,000 at m/z 200, and resolution for HCD spectra was set to 17,500 at m/z 200. Normalized collision energy was 30. The under fill ratio, which specified the minimum percentage of the target value likely to be reached at the maximum fill time, and was defined as 0.1%. The instrument was run with peptide-recognition mode enabled.

### Sequence database searching and data analysis

MS/MS spectra were searched using MASCOT engine (Matrix Science, London, UK; version 2.2) embedded into Proteome Discoverer 1.4 (Thermo Electron, San Jose, CA, USA) against the ovisaries sequence database (Uniprot_ovisaries_26933_20150114.fasta; 26933 sequences, released in Jan14th, 2015). For protein identification, qualitative analysis was performed using Proteome Discoverer 1.4 (Thermo, USA) software. All data were reported based on 98% confidence for protein identification as determined by false discovery rate (FDR) ≤1%. Isobaric Labeling Multiple File Distiller and Identified Protein iTRAQ Statistic Builder were used to calculate the ratios of protein, in which sample reference served as mixed internal parametal, based on the similarity weighted average of the intensities of report ions in each identified peptide. The final ratios were normalized with the median average protein ratio, assuming that most proteins remained unchanged in abundance. Protein identification was inferred from the unique peptide identification in the experiments. Statistical analysis was conducted that changed less than 1.2-fold was discarded.

### Bioinformatics analysis of differentially abundant proteins

Sequence information of the extracted the differentially proteins were retrieved from UniProtKB database (Version No: release2015_03) and conserve in FASTA format. The retrieved sequences were locally searched against Swiss-Prot Mammal by NCBI BLAST+ (ncbi-blast-2.2.28 + -win32.ext) software for sequence alignment. The functional annotation of homolog sequences was determined and transferred to the studied sequences according to similarity theory. The top 10 blast hits with E-value≤1e-3 for each query sequence were retrieved and loaded into Blast2GO (version 2.8.0) for GO mapping and annotation, in which the similarity of most sequences exceeded 81%. However, the sequences were un-annotated information by BLAST and without BLAST sequences were selected to retrieve the functional annotations of protein by InterProScan3 against EBI databases. Otherwise, the annotated information was supplemented further by Blast2GO in ANNEX to improve annotation accuracy. The GO project describing the function of proteins included three domains: biological process, molecular function and cellular component. Sequences were aligned against sheep protein sequence in Kyoto encyclopedia of genes and genomes (KEGG) GENES databases by using KEGG Automatic Annotation Server software, and annotation was mapped to annotated sequences and metabolic pathways in KEGG (https://www.genome.jp/kegg/) 15 based on KO codes.[Table t1-ab-24-0014]

### Quantitative real time polymerase chain reaction analysis and protein verification

Total RNA was extracted from WS and BS tissues by using RNAiso Plus (TaKaRa, Dalian, China) according to the manufacturer’s instructions, and transcribed into cDNA by using 5×PrimeScript RT Master Mix (Perfect Real Time) (TaKaRa, China). Gene primers were designed on the basis of a specific sequence through an online Primer 3 program, and cDNA was used for the quantitative real time polymerase chain reaction (qRT-PCR) analysis of mRNA abundance by using the gene specific forward and reverse primers. The transcript abundance of genes was quantified using the comparative threshold cycle method established by Livak.

Total protein was extracted from skin tissues, subjected to SDS-polyacrylamide gel electrophoresis (PAGE) and transferred onto nitrocellulose filter membranes (Boster, Wuhan, China). The membrane was blocked with 5% skimmed milk (Boster, China) in TBST (150 mM NaCl, 10 Mm Tris pH = 7.0, 0.05% Tween-20) for 2 h at room temperature, and then washed with TBST. The membrane was then incubated at 4°C overnight with polyclonal rabbit antibody and beta-actin (ACTB) (20536-1-AP; Protechtein, Wuhan, China, 1:4,000), fibrinogen alpha chain (FGA) (20645-1-AP; Protechtein, China; 1:1500), albumin (ALB) (16475-1-AP; Protechtein, China; 1:20,000), apolipoprotein A-1 (APOA1) (bs-0849R; Beijing Biosynthesis Biotechnology Co., Beijing, China; 1:1,000), amine oxidase (MAOA) (bs-6679R; Beijing Biosynthesis Biotechnology Co., China; 1:1,000), and glyceraldehyde-3-phosphate dehydrogenase (GAPDH) (Cat. No: bs-2188R; Beijing Biosynthesis Biotechnology Co., China; 1:3,000). Goat anti-rabbit IgG (γ-chain specific) antibody (1:5,000; Boster, China) was incubated with membrane for 1 h at 37°C. The membrane was washed and visualized using a super ECL Chemiluminescence plus kit (Boster, China). Band intensities were scanned and visualized using Image Lab software with a Bio-Rad system (Bio-Rad, USA). All protein contents were normalized to GAPDH level in each lane. Experiments were performed in triplicates.

### Immunofluorescence analysis

WS and BS tissues were fixed in Bouin solution, and paraffinized skin sections (8 μm) were deparaffinized. The prepared slides were dehydrated and blocked with 3% hydrogen peroxide (Boster, China) for 10 min, washed, and incubated with 5% bovine serum albumin (Boster, China) for 20 min at room temperature to block nonspecific binding. Afterwards, the sections were incubated in the presence of polyclonal rabbit antibody at 4°C, overnight. The next day, the sections were incubated in biotin conjugated DyLight-goat anti-rabbit IgG (Boster, China) at 37°C for 20 min (in this step, sections must be kept away from light). The sections were washed thrice (3 min each) with PBS and sealed with an anti-fade reagent (Boster, China).

### Statistical analysis

LC–ESI–MS/MS data were analyzed by searching the MASCOT engine (Matrix Science, London, UK; version 2.2) embedded into Proteome Discoverer 1.4 (Thermo Electron, San Jose, CA, USA) against the O. aries sequence database (Uniprot O. aries). GO functional annotation and data analysis was conducted on Blast2GO application software. All data were analyzed using SPSS statistical package version 17.0 (SPSS Inc., USA). All results were expressed as mean±standard deviation and analyzed using Student’s t-test and one-way analysis of variance.

## RESULTS

### Differential protein analysis between white skin and black skin sheep by iTRAQ

**Figure 1:**
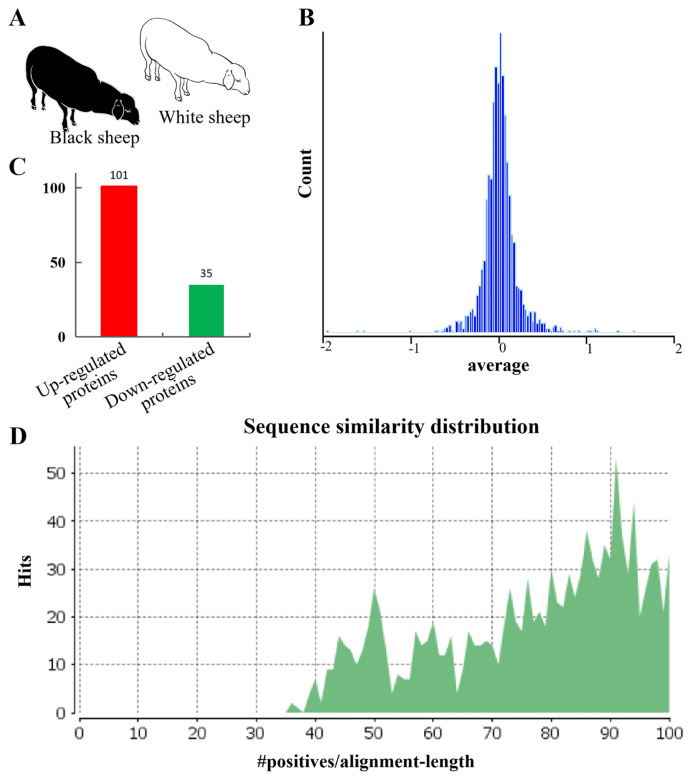
The results of iTRAQ in white and black sheep skin tissues. (A) The schematic diagram of white and black sheep. (B) The analysis of distribution ratio of quantitative histogram. (C) Statistical results of differential proteins in white and black sheep skin by iTRAQ technology. (D) Sequence alignment similarity distribution. iTRAQ, isobaric tags for relative and absolute quantification.

To identify proteins expressed in sheep skin, WS and BS protein libraries were analyzed by iTRAQ (Figure 1A). Figure 1B shows that the quantitative ratio of more proteins in WS and BS sheep approaches 0, whereas fewer proteins were distributed on both sides of the horizontal coordinate, which was the main differential protein for subsequent analysis and verification. A total of 136 differential proteins were selected based on the condition of ration >±1.2 and p<0.05 in WS and BS sheep. There were 101 up-regulated diferential proteins (Figure 1C). The sequence alignment analysis of differential protein sequences showed that the similarity of most target protein sequences exceeded 81%, indicating high reliability of the comparison results (Figure 1D).

Considering that the importance of differential protein was related with the differential fold, we listed 20 differential proteins, 13 of them were up-regulated and 7 were down-regulated. Table 2 lists the protein names and sequences in the Uniprot and Swiss-Prot databases, including the fold changes and t-test p-value of the protein. The up-regulated protein with difference ratio greater than 1.5 and p<0.05, and down-regulated protein with difference multiple less than 0.67 and p<0.05.

### GO classfication analysis of differential proteins in white skin and black skin sheep

**Figure 2:**
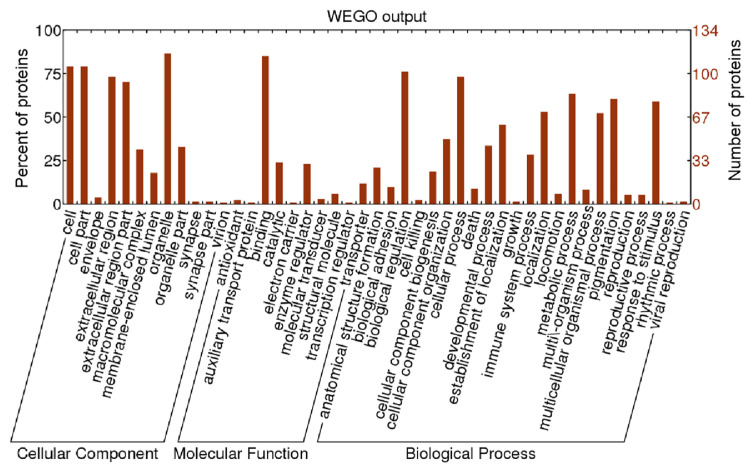
Classfication of gene ontology (GO) in the white and black sheep skin tissues.

The differential proteins screened were statistically analyzed from three aspects of biological process, molecular function through different sheep coat colors. The functional entries of target proteins primarily focused on biological processes through two functional annotations, and the most important one was enriched to pigmentation-function entries (Figure 2).

### GO slim analysis of differential proteins in white skin and black skin sheep

**Figure 3:**
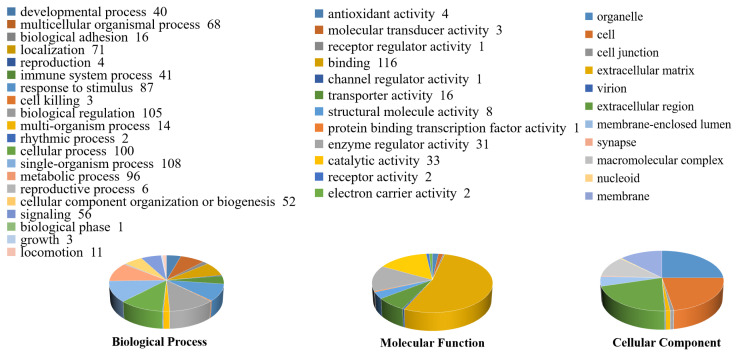
Statistics of gene ontology (GO) slim in the white and black sheep skin tissues.

As shown in Figure 3, the functions of differentially expressed proteins in sheep skin with different coat colors primarily focused on cell components and biological processes. Cell component was primarily reflected in the concentrated distribution of different proteins. Biological process was primarily reflected in the distribution of functional items of differential proteins. Molecular function involved a relatively smaller number and function items of protein distribution than cellular components and biological processes.

### Direct-interaction network map and validation analysis of differential proteins

**Figure 4:**
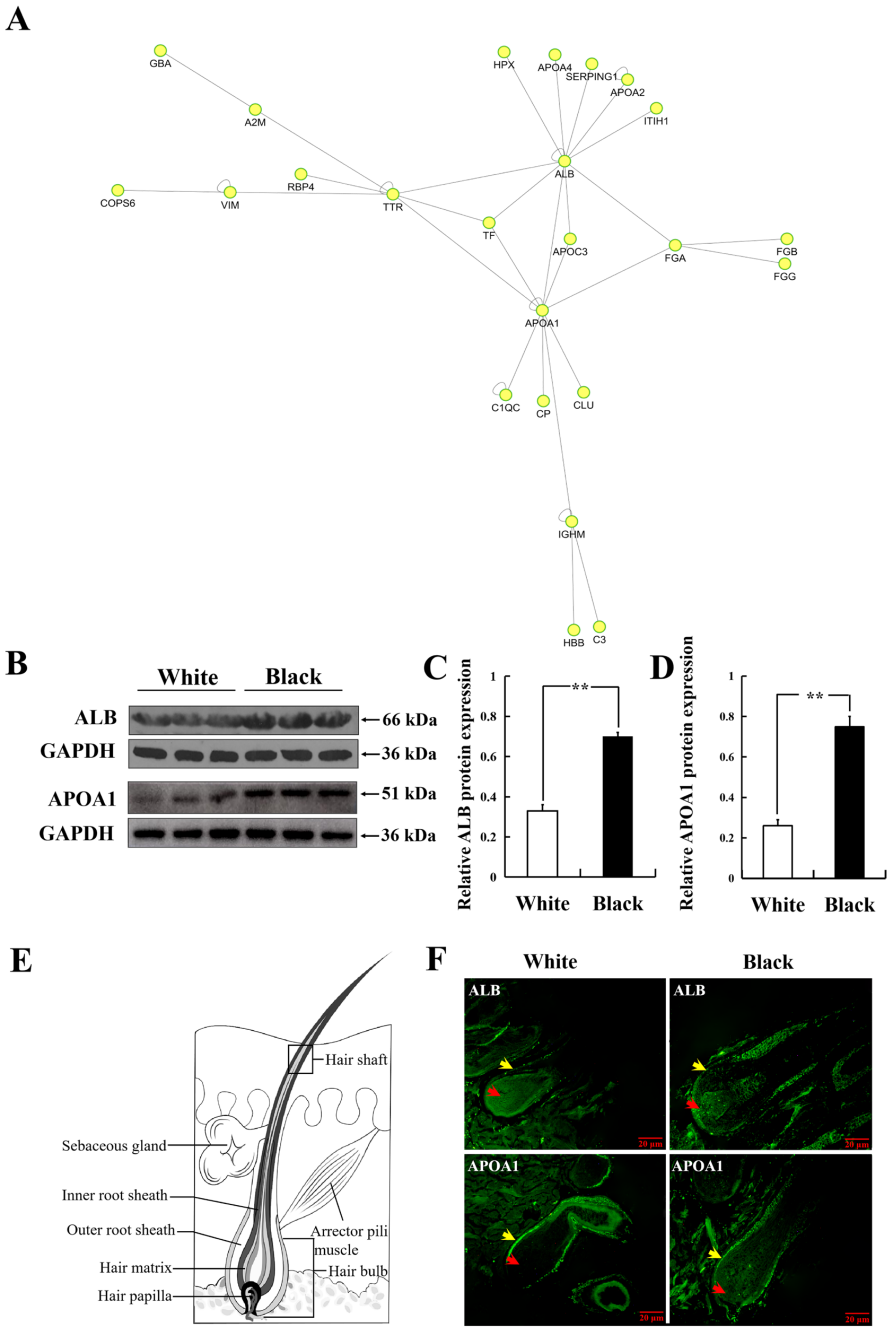
Direct interaction network map and validation analysis of differential proteins. (A) Interaction of differential proteins in the direct action network diagram. (B) Western blot results of ALB and APOA1 in white and black sheep skin. (C)–(D) Relative expression of ALB and APOA1 proteins statistical result. (E) Schematic diagram of hair follicle structure. (F) Distribution analysis of ALB and APOA1 expression in hair follicle of sheep by immunofluorescence analysis. ALB, albumin; APOA1, apolipoprotein A-1. ** p<0.01. The red arrow shows hair papilla; the yellow arrow shows outer root sheath.

Based on the gene symbol of the differentially expressed proteins, the interactions between the target proteins were determined by querying IntAct databases (Species: Mammalia), and interaction networks were generated using CytoScape software. Figure 4A (yellow nodes all represent directly interacting differential proteins) showed that A2M, transthyretin (TTR), vimentin (VIM), ALB, FGA, and APOA1 interacted with multiple proteins. Notably, ALB, TTR, APOA1, and FGA were characterized by critical differential proteins that interacted directly. The expression levels of ALB and APOA1 proteins were 1.57 and 1.91 folds in BS than that in WS sheep as determined by iTRAQ technology (Table 2). This finding indicated that ALB and APOA1 were large difference multiple in different coat color, and worthy of future study.

ALB and APOA1 proteins were detected by western blot. Figure 4B-4D shows that the protein expression level of ALB and APOA1 in BS sheep was significantly higher than that in WS sheep (ALB, APOA1: p<0.01). Results of immunofluorescence showed low expression of ALB protein in the outer root sheath of hair follicle in WS sheep (Figure 4F, yellow arrow). In the hair follicle of BS sheep, ALB protein was primarily expressed in hair papilla (Figure 4F, red arrow) and had low expression in the outer root sheath (Figure 4F, yellow arrow). APOA1 was primarily expressed in the outer root sheath of hair follicle in WS and BS sheep (Figure 4F, yellow arrow).

### KEGG signal pathway analysis of differential proteins in white skin and black skin sheep

**Figure 5:**
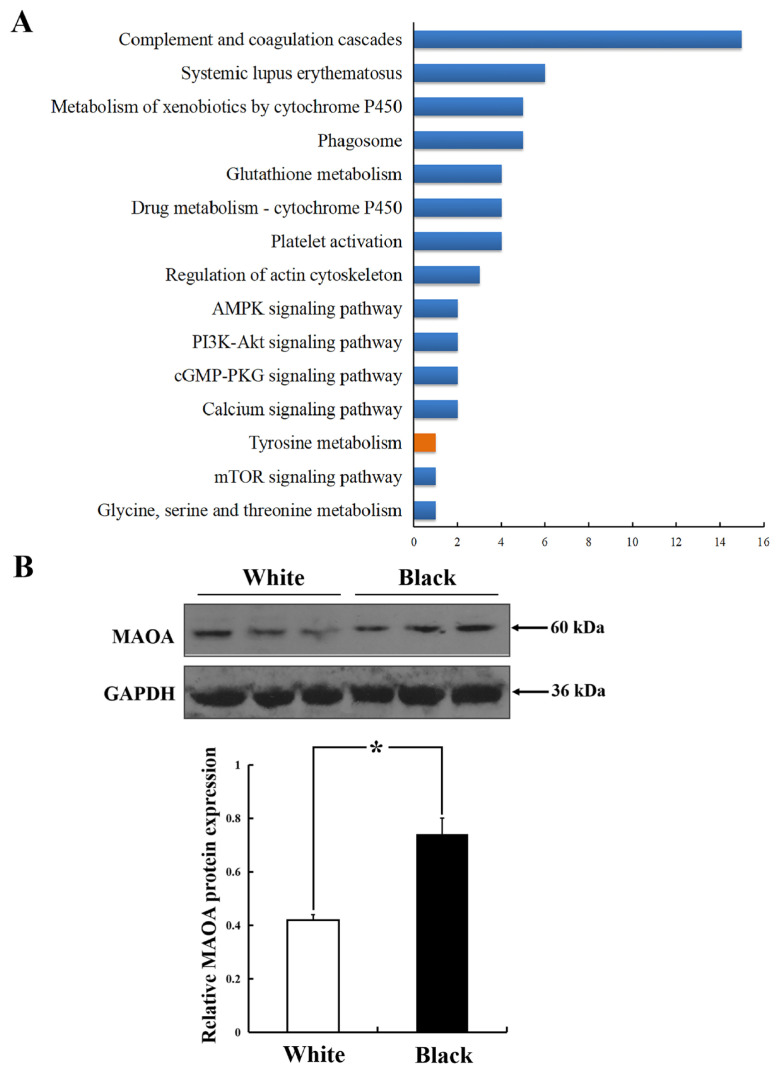
KEGG signal pathway and validation analysis of differential proteins in white and black skin tissues of sheep. (A) Distribution of differential proteins in KEGG signal pathway. (B) MAOA protein was detected by western blot. KEGG, Kyoto encyclopedia of genes and genomes; MAOA, amine oxidase. * p<0.05.

Target protein sequences identified from WS and BS sheep were compared with sheep protein sequences in the KEGG genes database and annotated to the relevant KEGG pathway by KO numbers of homologous/similar proteins. In this study, a total of 121 KEGG signaling/metabolic pathways associated with 55 differential protein sequences were identified. The distribution of the identified differential proteins in signaling pathways was statistically analyzed, and the results are shown in Figure 5A. The complement and coagulation cascade signaling pathways had 15 differential proteins. A high level of differential protein distribution existed in signaling, such as metabolism of xenobiotics by cytochrome P450 (ko00980) and platelet activation (ko04611). Combined with our research background analysis, the tyrosine metabolism (ko00350) was extracted, which is the downstream pathway of melanogenesis pathway (ko04916). This finding indicated that detection and appraisal by LC-MS/MS technique had certain reliability.

The tyrosine metabolism pathway was screened by MAOA differential protein. MAOA differential protein was enriched in the downstream pathway of melanogenesis, and we speculated that MAOA was related to the formation of coat color. Therefore, further verification analysis was conducted by western blot. As shown in Figure 5B, the expression level of MAOA protein in BS sheep was significantly higher than that in WS sheep (p<0.05), consistent with the iTRAQ results.

### Analysis of ACTB and FGA expression in sheep skin with different coat colors

Four differential proteins were enriched in platelet activation (ko04611) pathway by KEGG analysis. ACTB was a down-regulated proteins and FGA was an up-regulated proteins according to differential protein analysis. The expression of ACTB and FGA proteins was 0.81 and 1.65 folds in BS compared with that in WS of sheep by iTRAQ technology. However, ACTB and FGA proteins may be related closely to melanin synthesis according to literature.

**Figure 6:**
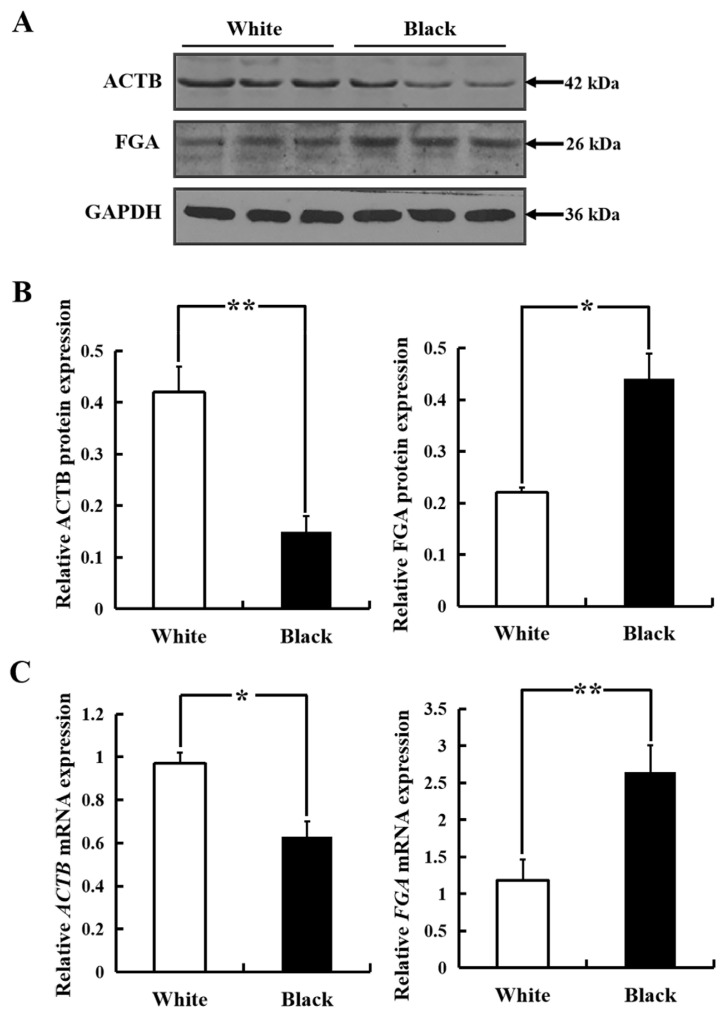
Analysis of ACTB and FGA expression in sheep skin with different coat colors. (A) Western blot results of ACTB and FGA in white and black sheep skin. (B) Relative protein expression levels of ACTB and FGA in white and black sheep skin. (C) Relative expression of *ACTB* and *FGA* mRNA in skin samples collected from white and black sheep. *ACTB*, beta-actin; *FGA*, fibrinogen alpha chain. * p<0.05, ** p<0.01.

The expression of ACTB protein in WS sheep tissue was significantly higher than that in BS sheep tissue (p<0.05) (Figure 6A, 6B). The relative expression level of FGA protein in BS sheep was higher than that WS sheep (p<0.05). The *ACTB* and *FGA* mRNA expression level was consistent with the protein level from each coat color (Figure 6C).

### Distribution analysis of ACTB and FGA expression in hair follicle of sheep

**Figure 7:**
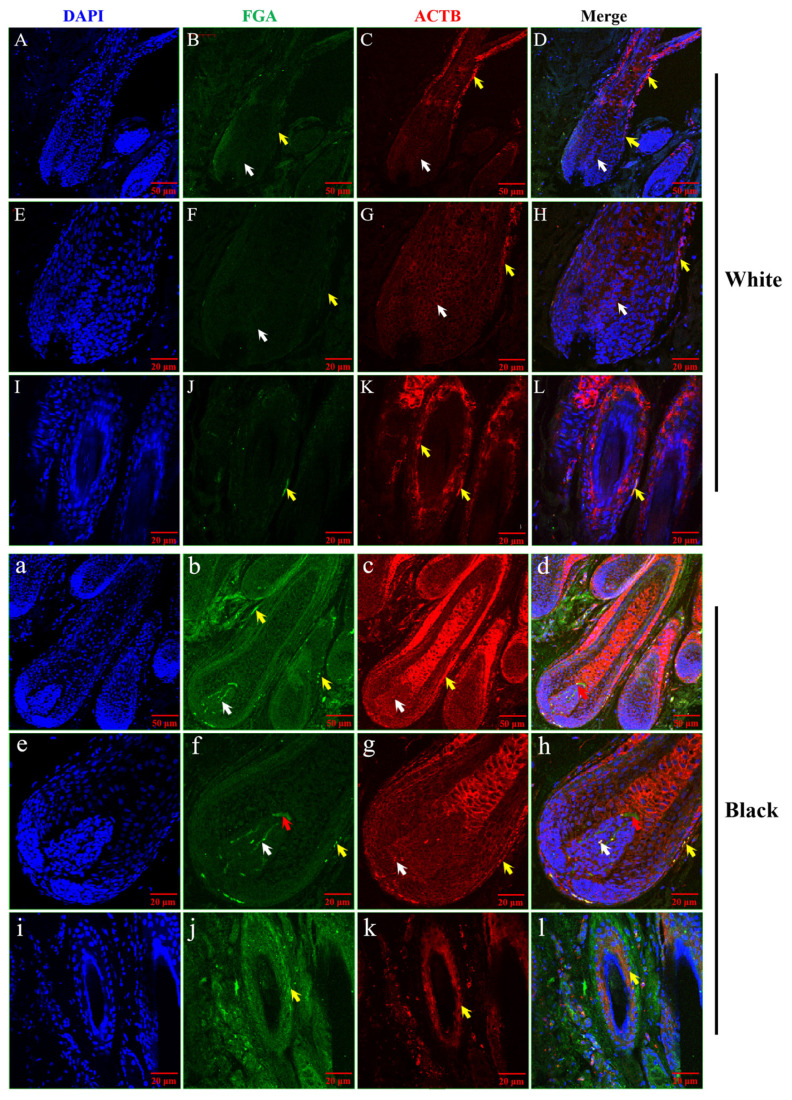
Distribution analysis of ACTB and FGA expression in hair follicle of sheep by laser confocal microscopy. (A), (B), (C) Positive DAPI, FGA, and ACTB expression in hair follicle in white sheep skin. (D) The superposition of positive DAPI, FGA, and ACTB expression in hair follicle in white sheep skin. (E), (F), (G) Positive DAPI, FGA, and ACTB expression in hair papilla in white sheep skin. (H) The superposition of positive DAPI, FGA, and ACTB expression in hair papilla in white sheep skin. (I), (J), (K) Positive DAPI, FGA, and ACTB expression in outer root sheath in white sheep skin. (L) The superposition of positive DAPI, FGA, and ACTB expression in outer root sheath in white sheep skin. The white arrow shows hair papilla; the yellow arrow shows outer root sheath. (a), (b), (c) Positive DAPI, FGA, and ACTB expression in hair follicle in black sheep skin. (d) The superposition of positive DAPI, FGA, and ACTB expression in hair follicle in black sheep skin. (e), (f), (g) Positive DAPI, FGA, and ACTB expression in hair papilla in black sheep skin. (h) The superposition of positive DAPI, FGA, and ACTB expression in hair papilla in black sheep skin. (i), (j), (k) Positive DAPI, FGA, and ACTB expression in outer root sheath in black sheep skin. (l) The superposition of positive DAPI, FGA, and ACTB expression in outer root sheath in black sheep skin. The white arrow shows hair papilla; the yellow arrow shows outer root sheath. ACTB, beta-actin; FGA, fibrinogen alpha chain; DAPI, 4',6-Diamidino-2-phenylindole.

Distribution analyzed of ACTB and FGA proteins were observed expression in the hair follicle of white and black sheep by laser confocal. In the hair follicles of WS sheep (Figure 7B and 7F, white arrow), FGA protein was almost not expressed in the dermal papilla of white hair follicle, and a small amount was expressed in the outer root sheath of hair follicle (Figure 7J, yellow arrow). ACTB protein was expressed around the cells of the dermal papilla of hair follicle (Figure 7C and 7G, white arrow). ACTB protein was obviously expressed in the outer root sheath of hair follicle (Figure 7C and 7D, yellow arrow).

In hair follicles of BS tissue (Figure 7b and 7f), FGA protein was significantly expressed in the dermal papilla of hair follicle and also in the hair matrix (Figure 7f, red arrow). FGA protein was also expressed in the outer root sheath of hair follicle (Figure 7b and 7j, yellow arrow). ACTB protein was expressed in the dermal papilla of hair follicle, but not in hair matrix (Figure 7f, white arrow). As shown in Figure 7c and 7k (yellow arrow), ACTB protein was strongly expressed in the outer root sheath of hair follicle compared with FGA protein.

## DISCUSSION

Wool maintains body temperature and protects and also has unique economic value. Consumers pursue wool-related products and bring economic benefits to the wool market. As a wool-producing animal, wool color is the main economic and phenotypic trait of sheep [16], which used as a genetic marker for individual identification, blood system, and breed affiliation. Therefore, studying the formation mechanism of different coat colors has great significance for improving the economic traits and genetic breeding of sheep. The color of wool depends primarily on the composition, quantity, and distribution of melanin [17]. Notably, the coat color of different phenotypes was determined by melanin, which depends on the interaction of different genes.

Quantitative proteomics is extensively applied to detect differential proteins and pathways in many kinds of genetic phenotypic analysis. In the current work, a 6-plex iTRAQ analysis enabled the investigation of large-scale protein alterations. A total of 1,704 proteins were identified, and 136 proteins were found to be differentially expressed; 101 and 35 were up-regulated and down-regulated, respectively. The melanin pigmentation of mammals is regulated by a number of factors at the systemic, tissue, cellular, and subcellular levels [18]. The differential proteins were statistically analyzed from three aspects: biological process, molecular function and cell component through two functional annotations of the target proteins, including cellular process, metabolic process, catalytic activity, molecular transducer activity, cell junction extracellular matrix, and so on. Furthermore, pigmentation function entry was enriched in the bioloical process. Similarly, skin transcriptome profiles associated with coat color in sheep, which indicated that the majority of the GO terms including pigmentation enriched in the differentially expressed genes [11]. The pigmentation entry is related to coat color formation. This analysis improved the accuracy of the annotation of functional items of target proteins. KEGG results showed that 121 signaling pathways involving 55 different proteins were extracted. Platelet activation and tyrosine metabolism pathway were significant. Unfortunately, the melanogenesis pathway was not extracted in this proteomic analysis. However, the tyrosine metabolism pathway that is the down pathway of melanogenesis was extracted. Numerous of studies have confirmed that tyrosine was closely related to pigment synthesis, which is a key factor regulating the formation of coat color, including that of sheep [19–21]. The interaction network analysis diagram showed that among the identified target proteins, A2M, TTR, VIM, ALB, FGA, FGB (Fibrinogen beta chain), FGG, C1QC, and APOA1 were involved in the direct interaction of proteins. ALB, APOA1, and FGA proteins have been discovered to be related to coat color of sheep, and further research is required to explore their correlation with coat color.

The differential protein interaction analysis pointed out that ALB and APOA1 proteins played pivotal roles according to iTRAQ. Further studies conclusively demonstrated that the expression level of ALB protein was higher in BS than that in WS. Morphological analysis revealed that ALB protein was mainly distributed in the hair papilla that distributed blood vessel. At present, the involvement of ALB in melanin synthesis and coat color regulation primarily focuses on two aspects. On one hand, ALB and vitamin-D binding protein which is a paralog of the mammalian endothelin receptor B (*EDNRB*) gene [22]. EDNRB is responsible for coat color formation in mammals [23,24], and ALB may affect melanin formation by playing a EDNRB-like role. On the other hand, ALB can control the size of dopamine-eumelanin aggregates formed in Dopa solutions upon oxidation [25]. Dopa provides the starting material for melanin biosynthesis in all animals [26]. Combined with our experimental results, the above findings suggested that ALB was associated with coat color formation in sheep.

Apolipoprotein A-1 (APOA1), the primary protein constituent of high density lipoprotein, exists in blood. APOA1 was differentially expressed protein in WS and BS sheep based on iTRAQ technology, confirming that the expression level of APOA1 protein was higher in BS than that in WS. Notably, immunofluorescence results showed that APOA1 was strongly expressed in the outer root sheath of hair follicles in white and black sheep, whereas almost no expression was found in dermal papilla. The stem cell pool of human hair follicles’ outer root sheath transform into functional melanocytes [27]. Melanocyte (melanocyte precursor) is primarily exists in the outer root sheath of the hair follicle, so we conjectured that APOA1 affected the production of melanocyte precursors to melanoblasts and resulted in different coat colors. At present, APOA1 has not been reported to be associated with melanin synthesis and coat color formation. However, considering our experiment results, we put forward provisionally that APOA1 is perhaps related to the coat color formation of sheep.

Tyrosine metabolism pathway regulates the formation of melanin, include eumelanin and pheomelanin synthesis. Although numerous enzymatic catalyzed and chemical reactions are involved in melanogenesis process, the enzymes such as tyrosinase and tyrosinase-related proteins played a major role in melanin synthesis [28]. Thus, target protein focus on MAOA of tyrosine metabolism pathway. In the present study, the expression of MAOA protein was higher in BS tissue sheep than that in WS tissue of sheep, and suggested that MAOA was expressed differently in different coat colors. A previous research has reported that an imbalance in Dopa metabolism leads to the down regulation of MAOA [29]. Furthermore, tyrosinase also catalyzes the oxidation of Dopa to Dopa quinone, a precursor of melanin [30]. TYR and MAOA are significantly differential genes associated with iris colors by genome-screening [31]. Accordingly, we speculated that MAOA affected melanin synthesis through the tyrosine metabolism pathway, which is related to the coat color formation of sheep.

Platelet activation is another signaling pathway enriched in four different proteins. Tyrosine kinase inhibitor with antiangiogenic activity through targeting platelet-derived growth factor receptors and effected on hair pigmentation in mice [32]. We selected ACTB and FGA proteins and detected and analyzed them to prove that the relative expression of ACTB protein in WS tissue of sheep was significantly higher than that in BS tissue. ACTB was reportedly not a reliable in melanocytes cells on account of their lower expression stability, consistent with our results. Analogously, ACTB was melanocyte lineage-specific genes in vitiligo lesion needle biopsies and to predict the occurrence of perifollicular repigmentation in depigmented macules [33]. Including, ACTB as a hub protein associated with tyrosinase-mediated melanogenesis in melanoma cells [34]. The research results suggested that ACTB was involved in the formation of melanin in melanocyte or melanoma cells. Combined with our experimental results, ACTB expression was different in white and black sheep skin, and proposed ACTB protein is unsuitable for housing gene or protein in the study of hair color and pigmentation. FGA is the downstream factor of ACTB in platelet activation and ACTB inhibits FGA expression according to KEGG analysis. The relative expression of FGA protein in BS tissue was significantly higher than that in WS tissue. Moreover, FGA protein was mainly expressed in the outer root sheath, dermal papilla and hair matrix in hair follicles of black sheep. In hair follicle, melanin is generated inside melanocytes and then is delivered to epidermal keratinocytes by the dendrites of melanocytes for epidermal pigmentation. Therefore, we conjectured that ACTB and FGA proteins of the platelet activation signaling pathway may be involved in formation of coat color in sheep, but the molecular mechanism needs further study.

## CONCLUSION

Taken together, to our knowledge this is the first report of proteomics analysis of sheep skin from animals with white and black coat color. The present studies have described and revealed a set differentially expressed novel proteins in sheep skin, which potentially related to coat color and other physiological functions. The 5 novel proteins and 2 signal pathway exclusively expressed in skin of sheep with black coat color, which are the particular interest for further studies to elucidate their functional roles in coat color regulation. Results are foundational for future studies to potentially manipulate coat color via pharmacological and genetic approaches.

## Figures and Tables

**Table 1 t1-ab-24-0014:** Primer information for target and housekeeping genes

Gene	Primer sequences (5'→3')	product size (bp)	Tm (°C)
*ACTB*	F: CCCTGGAGAAGAGCTACGAG	131	58.9
	R: GGTAGTTTCGTGAATGCCGC		
*FGA*	F: CCCTGGAGAAGAGCTACGAG	125	59.0
	R: GGTAGTTTCGTGAATGCCGC		
*18S rRNA*	F: GAAGGGCACCACCAGGAGT	158	60.0
	R: CAGACAAATCACTCCACCAA		

*ACTB*, beta-actin; *FGA*, fibrinogen alpha chain; F, Sense primers; R, Antisense primer.

**Table 2:** Parts differentially expressed proteins of white and black sheep skin by iTRAQ technology

Items	Accession	Description	Unique peptides	Average Black/White	t-test p-value
Up-regulated proteins	W5QH46	Uncharacterized protein OS=Ovis aries GN=KNG1 PE=4 SV=1 - [W5QH46_SHEEP]	6	1.50	0.00055
	W5P246	Uncharacterized protein (Fragment) OS=Ovis aries GN=TM9SF2 PE=4 SV=1 - [W5P246_SHEEP]	2	1.54	0.00130
	P14639	Serum albumin OS=Ovis aries GN=ALB PE=1 SV=1 - [ALBU_SHEEP]	2	1.57	0.00036
	W5NQ46	Fibrinogen beta chain OS=Ovis aries GN=FGB PE=4 SV=1 - [W5NQ46_SHEEP]	12	1.57	0.00035
	Q7M371	Plasma proteinase inhibitor (Fragment) OS=Ovis aries PE=1 SV=1 - [Q7M371_SHEEP]	1	1.60	0.00094
	W5Q5H8	Fibrinogen alpha chain OS=Ovis aries GN=FGA PE=4 SV=1 - [W5Q5H8_SHEEP]	22	1.62	0.00024
	P02075	Hemoglobin subunit beta OS=Ovis aries GN=HBB PE=1 SV=2 - [HBB_SHEEP]	4	1.67	0.00314
	W5PSQ7	Uncharacterized protein OS=Ovis aries PE=4 SV=1 - [W5PSQ7_SHEEP]	8	1.77	0.00387
	W5NX51	Uncharacterized protein OS=Ovis aries GN=APOA1 PE=4 SV=1 - [W5NX51_SHEEP]	14	1.91	0.00036
	W5NXW9	Uncharacterized protein (Fragment) OS=Ovis aries GN=IGHM PE=4 SV=1 - [W5NXW9_SHEEP]	11	2.09	0.00012
	W5PWE9	Serum albumin OS=Ovis aries GN=ALB PE=4 SV=1 - [W5PWE9_SHEEP]	1	2.16	0.00011
	A2P2I3	VH region (Fragment) OS=Ovis aries GN=VHPE=2 SV=1 - [A2P2I3_SHEEP]	2	2.21	0.00532
	W5QHZ5	Uncharacterized protein OS=Ovis aries PE=4 SV=1 - [W5QHZ5_SHEEP]	4	2.95	0.00003
Down-regulated proteins	P02076	Hemoglobin subunit beta OS=Ovis orientalis musimon GN=HBB PE=1 SV=1 - [HBB_OVIMU]	2	0.23	0.00375
	W5QGJ1	Uncharacterized protein (Fragment) OS=Ovis aries GN=GOLT1B PE=4 SV=1 - [W5QGJ1_SHEEP]	1	0.32	0.00157
	W5Q6G0	Uncharacterized protein OS=Ovis aries GN=LOC101111440 PE=3 SV=1 - [W5Q6G0_SHEEP]	1	0.34	0.04442
	W5PE20	Uncharacterized protein (Fragment) OS=Ovis aries GN=PELP1 PE=4 SV=1 - [W5PE20_SHEEP]	1	0.61	0.03130
	W5NWS0	Uncharacterized protein (Fragment) OS=Ovis aries PE=4 SV=1 - [W5NWS0_SHEEP]	1	0.64	0.00255
	W5P994	Lon protease homolog, mitochondrial OS=Ovis aries GN=LONP1 PE=3 SV=1 - [W5P994_SHEEP]	2	0.66	0.04231
	W5PF02	Uncharacterized protein OS=Ovis aries GN=SRPRB PE=4 SV=1 - [W5PF02_SHEEP]	1	0.66	0.02965

iTRAQ, isobaric tags for relative and absolute quantification.
